# Editorial Responsibilities

**Published:** 1885-10

**Authors:** 


### EDITORIAL RESPONSIBILITIES.

The question of the extent to which editorial supervision of the communications of correspondents should be exercised is a vexed one. Occasionally, a generally well informed man falls into egregious errors in writing on a subject with some phases of which he is not familiar. Shall the editor take the liberty of correcting such mistakes, or shall he let his correspondent exhibit his ignorance? Many writers are extremely sensitive concerning any editorial liberties with their manuscript, and resent even verbal alterations, made by one who is supposed to be an expert in the use of written language. Yet such changes are sometimes essential to explicitness. Some editors shirk all responsibility by a standing notice that the journal is not responsible for the expression of opinions by correspondents. But if any writer is allowed, without protest, to present absolutely false teachings, few readers will hold the editor entirely blameless. Upon a debatable question the utmost liberty should undoubtedly be allowed, but on vital questions of fact, what is the editorial duty?

In the September number of one of our dental journals, for instance, a correspondent is allowed to say concerning nitrous oxide: "When breathed, it produces anæsthesia by depriving the blood of oxygen, and causes sensations similar to those experienced in drowning."

This, in effect, is the declaration that the anæsthesia of nitrous oxide is nothing but asphyxia, an assertion than which nothing could be more false. Such teachings are dangerously erroneous, but does the proper "editing" of manuscript justify the correcting of such misconception? If so, then the editor becomes the arbitrary censor of all that comes under his hand, and his journal will be likely to reflect only his own opinions. If, on the other hand, his duties extend only to the correcting of grammatical, orthographical, or typographical errors, and to the general euphonistic presentation of papers, he is likely to publish an infinite deal of misconception and untruth. It is a vexed question, and the editor who escapes censure, by either writer or reader, is possessed of extraordinary tact, or is unusually fortunate.

There is another phase of the question: Shall an editor permit flagrant and known plagiarisms by his correspondents? The instances of willful cribbing from others, of the presentation as original of whole pages bodily stolen, are not infrequent. Sometimes these literary thefts are from sources probably unknown to the editor, and in such cases he is, of course, blameless.

An instance of this is found in a paper on anæsthesia, read before the Minnesota Dental Society, and published in one of our journals. It had a strangely familiar sound, and on reference to a paper read by us before the American Dental Association, at its meeting in Boston, in 1880, and afterwards published in pamphlet form, we find an identity in thought and expression that is too complete to be the result of mere coincidence. Whole paragraphs are, word for word, the same in both articles. Let any one compare the paper as published in *The Dental Register*, with the one in the Transactions of the A. D. A. for 1880, and note the something more than similarity. As our article had the advantage of five years in its presentation, we feel that we cannot be charged with having borrowed it from Dr. Jenison. We rejoice that what we said made so deep an impression upon one mind, but we wish that for his own credit the author had made some explanation of the identity of both thought and expression.

Of course no editor can be held responsible for the presentation of a literary piracy unless he has knowledge of it, but what is he to do in cases of absolute misconception and misstatement of scientific facts?

